# Adverse drug events associated with ibrutinib for the treatment of elderly patients with chronic lymphocytic leukemia: A systematic review and meta-analysis of randomized trials

**DOI:** 10.1097/MD.0000000000016915

**Published:** 2019-08-16

**Authors:** Yanhua Zhou, Hongtao Lu, Meifeng Yang, Chenhong Xu

**Affiliations:** a Department of Hematology, Jingzhou First People's Hospital, The First Affiliated Hospital of Yangtze University, Jingzhou; b Department of Cardiology, Jingzhou Central Hospital, The Second Clinical Medical College, Yangtze University, Jingzhou, Hubei, People's Republic of China.

**Keywords:** adverse drug events, anemia, blood disorders, chronic lymphocytic leukemia, neutropenia, thrombocytopenia

## Abstract

**Background:**

Chronic lymphocytic leukemia (CLL) is a rare hematological malignancy classified in the non-Hodgkin's lymphoma category. Ibrutinib, a first-in-class Bruton tyrosine kinase inhibitor has been approved for use in the treatment of CLL. This drug has shown beneficial effects including a higher overall response rate, sustained remissions, and a tolerable toxicity level. In this meta-analysis, we aimed to compare the adverse drug events which were associated with the use of ibrutinib for the treatment of patients with CLL.

**Methods:**

A careful search was carried out through the Cochrane Central, EMBASE, MEDLINE (PubMed), and through www.ClinicalTrials.com. The following criteria for inclusion were considered: Both randomized trials and observational cohorts; Studies comparing the adverse drug events observed with the use of ibrutinib versus a control group for the treatment of CLL. The RevMan software (version 5.3) was used to carry out this analysis and the analyzed data were represented by risk ratios (RR) and 95% confidence intervals (CI).

**Results:**

A total number of 2456 participants with CLL were included in this analysis. One thousand one hundred thirteen participants were treated with ibrutinib whereas the remaining 1343 participants were assigned to the control (non-ibrutinib) group. Results of this current analysis showed Ibrutinib not to be associated with significantly higher risk of anemia (RR: 0.90, 95% CI: 0.67–1.21; *P* = .49), thrombocytopenia (RR: 0.61, 95% CI: 0.32–1.14; *P* = .12), neutropenia (RR: 0.50, 95% CI: 0.25–1.00; *P* = .05), and febrile neutropenia (RR: 0.89, 95% CI: 0.32–2.49; *P* = .83) in these patients with CLL. The risk for respiratory tract infection was also similarly manifested (RR: 1.01, 95% CI: 0.78–1.30; *P* = .96). However, ibrutinib was associated with a high risk of abdominal manifestations in comparison to the control group (RR: 1.62, 95% CI: 1.32–2.00; *P* = .00001). The risk for diarrhea was also significantly higher in the Ibrutinib group (RR: 2.14, 95% CI: 1.44–3.17; *P* = .0002).

**Conclusions:**

During the treatment of CLL, ibrutinib was not associated with significantly higher risks of anemia, thrombocytopenia, or neutropenia compared to the control group. However, abdominal manifestations were significantly higher with ibrutinib. Advanced phase trials should further confirm this hypothesis.

## 1 Introduction

Chronic lymphocytic leukemia (CLL) is a rare hematological malignancy classified in the non-Hodgkin's lymphoma category.^[1]^ This blood disorder is mainly diagnosed in elderly male patients, and it progresses slowly with a median survival time period of 10 years.^[1]^ The exact cause of this malignancy is unknown; however, the risk factors^[2]^ for the occurrence of CLL are: old age, race (Whites), family history of blood and bone marrow tumors, and exposure to certain chemical substances including benzene, herbicides, and insecticides/pesticides.^[3]^

Nowadays, use of higher quantities of herbicides in vegetable fields, use of excess amounts of pesticides^[4,5,6]^ for protection and production of better quality vegetables in the market might be one of the causes resulting in an increase in the total number of CLL patients worldwide.

Ibrutinib, a first-in-class Bruton tyrosine kinase inhibitor has been approved by the Food and Drug Administration for the treatment of CLL in the United States.^[7,8]^ This drug has shown beneficial effects including a higher overall response rate, sustained remissions, and a tolerable toxicity level.^[9]^ Nevertheless, the adverse drug events of ibrutinib have seldom been systematically assessed.

In this meta-analysis, we aimed to compare the adverse drug events which were associated with the use of ibrutinib in comparison to a control group for the treatment of patients with CLL.

## 2 Methods

### 2.1 Searched databases and searched strategies

A careful search was carried out through the Cochrane Central, EMBASE, MEDLINE (PubMed), and through www.ClinicalTrials.com. Reference lists of relevant articles were also further explored for suitable publications.

The following searched terms were used in the above-mentioned searched databases:

(1) Ibrutinib and chronic lymphocytic leukemia;
(2) Ibrutinib and CLL;
(3) Ibrutinib and leukemia;
(4) Ibrutinib, chronic lymphocytic leukemia, and drug events;
(5) New therapy and chronic lymphocytic leukemia;
(6) Imbruvica and chronic lymphocytic leukemia.

This search process was restricted only to English publications.

### 2.2 Criteria for inclusion and exclusion

The following criteria for inclusion were considered:

(1) Both randomized trials and observational cohorts;
(2) Studies comparing the adverse drug events which were associated with the use of ibrutinib versus a control group for the treatment of CLL.

The following criteria for exclusion were considered:

(1) Review articles including systematic reviews, meta-analyses, and case reports;
(2) Publications that did not include patients with CLL;
(3) Publications that showed the adverse drug events which were associated with ibrutinib but without comparison with a control group;
(4) Publications that did not report the correct endpoints;
(5) Repeated publications (studies that were repeatedly obtained through search databases).

### 2.3 Data extraction and quality assessment

After having carefully read the relevant publications, data were independently extracted by 4 authors. Data included the detailed total number of CLL participants in the experimental and control groups, respectively, the reported type of study, other general, and baseline properties of the studies including enrollment time frame of the participants, age, gender, and the duration of therapy.

Any disagreement or confusion which if present, was resolved by the corresponding author.

The methodological quality of the trials was assessed with reference to the criteria suggested by the Cochrane collaboration.^[10]^ During the assessment of the methodological quality, grades were allotted to represent a low (grade A), moderate (grade B), or high (grade C) risk of bias.

### 2.4 Statistical analysis

The RevMan software (version 5.3) was used to carry out this analysis. The analyzed data were represented by risk ratios (RR) and 95% confidence intervals (CI).

Meta-analyses are often associated with heterogeneity. The *Q* statistic test (in probability) and the *I* ^2^ statistic test (in percentage) were used to assess for heterogeneity. A *P*-value less than .05 was considered as statistically significant. Concerning the *I* ^2^ statistic test, a high heterogeneity was represented by a higher value of *I* ^2^ whereas a lower *I* ^2^ value denoted a lower heterogeneity.

A random statistical model was used for this analysis.

Sensitivity analysis was also carried out (exclusion of each study by turn to carry out a new analysis each time).

Since this analysis consisted of a small volume of studies, observation through funnel plots could easily assess for publication bias.

### 2.5 Ethical approval

Since this analysis did not involve human or animal experiments performed by any of the authors, ethical or board review approval was not required.

## 3 Results

### 3.1 Searched outcomes

A total number of 835 publications were searched from electronic databases (PRISMA guidelines).^[11]^

The titles and abstracts were carefully assessed by the authors, and, falling outside the scope of this research title, a list of 748 publications were directly eliminated. Eighty-seven full-text articles were assessed based on the criteria of inclusion and exclusion.

Further studies were eliminated: literature reviews (2); case reports (3); pooled analyses (2); did not report the required endpoints (4); included patients with other blood disorders without including CLL participants (8); did not involve a control group (9); were based on indirect comparisons (4); duplicated studies (50).

**Figure 1:**
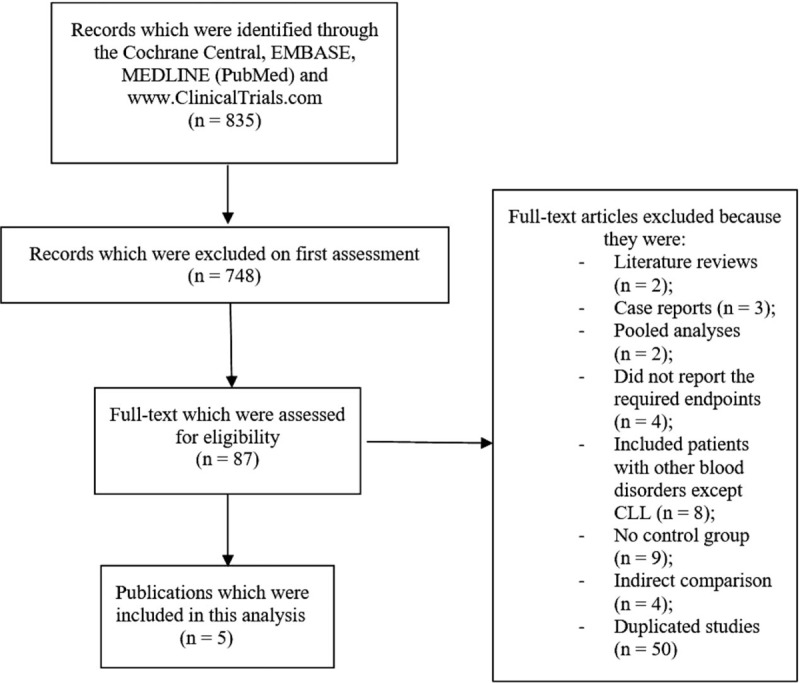
Flow diagram showing the study selection for inclusion in this meta-analysis.

Finally, only 5 studies^[12,13,14,15,16]^ were selected for this analysis as shown in Figure 1.

### 3.2 General properties of the studies

Listed in Table 1 are the general features of the trials in this analysis. As shown in Table 1, all the studies were randomized trials which enrolled participants with CLL from the year 2008 to the year 2016. A total number of 2456 participants with CLL were included in this analysis. One thousand one hundred thirteen participants were treated with ibrutinib whereas the remaining 1343 participants were assigned to the control (non-ibrutinib) group.

**Table 1:**
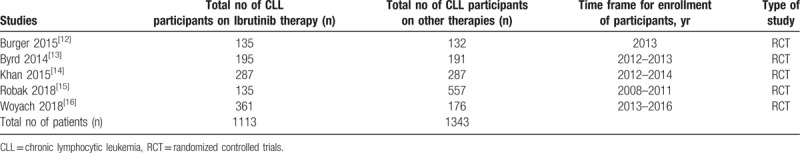
General features of the studies.

After the methodological quality assessment of the trials, a grade A to B was assigned representing low to moderate risk of bias.

### 3.3 Outcomes which were reported

The adverse drug outcomes which were reported in each study have been given in Table 2. Based on the most common outcomes which were reported in majority of the studies, the following endpoints were assessed:

(1) Anemia;
(2) Thrombocytopenia;
(3) Neutropenia;
(4) Febrile neutropenia;
(5) Respiratory tract infections;
(6) Any infection;
(7) Abdominal manifestations including vomiting, abdominal pain, constipation, and diarrhea;
(8) Arthralgia;
(9) Hypertension;
(10) Peripheral edema;
(11) Diarrhea.

**Table 2:**
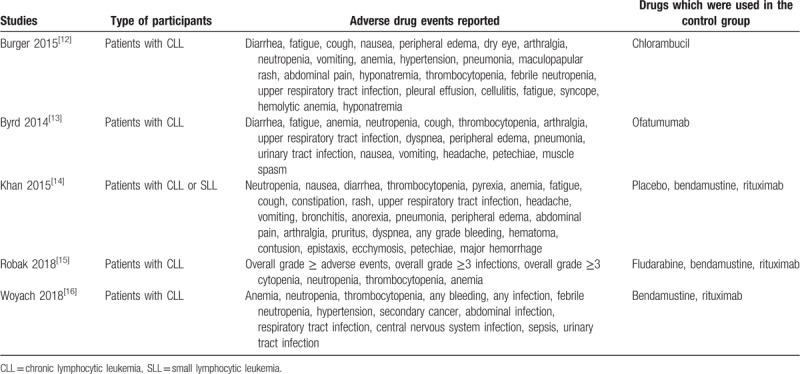
Adverse drug events which were reported.

The drugs which were used in the control group have also been listed in Table 2.

### 3.4 Baseline features of the CLL participants

Tables 3 and 4 report the baseline properties of the CLL participants.

**Table 3:**
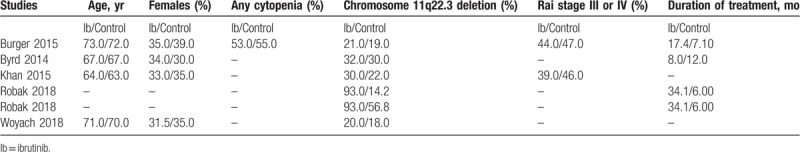
Baseline features of the participants (per individual study) with chronic lymphocytic leukemia.

**Table 4:**
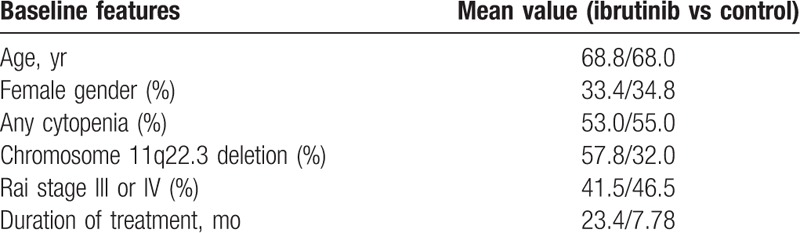
Baseline features (average value) of the participants with chronic lymphocytic leukemia.

Mean age of the CLL participants varied from 63.0 to 73.0 years. Majority of the CLL participants were males, and the average percentage of female participants was 33.4% to 34.8%. Chromosome 11q22.3 deletion varied from 14.2% to 93.0%, and the duration of treatment varied between 6.0 and 34.1 months. The mean value for each group has been listed in Table 4.

### 3.5 Main results of this analysis involving CLL participants

**Figure 2:**
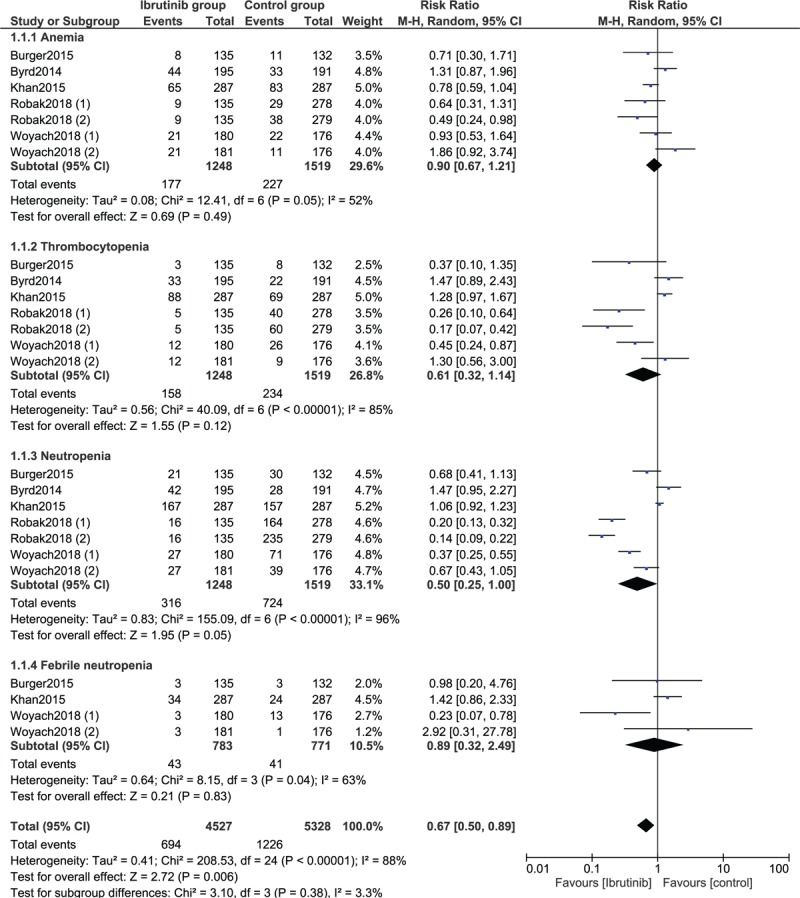
Forest plot demonstrating the adverse drug events observed with ibrutinib versus the control group for the treatment of patients with chronic lymphocytic leukemia (Part I).

Results of this current analysis showed ibrutinib not to be associated with significantly higher risk of anemia (RR: 0.90, 95% CI: 0.67–1.21; *P* = .49), thrombocytopenia (RR: 0.61, 95% CI: 0.32–1.14; *P* = .12), neutropenia (RR: 0.50, 95% CI: 0.25–1.00; *P* = .05), and febrile neutropenia (RR: 0.89, 95% CI: 0.32–2.49; *P* = .83) in these patients with CLL as shown in Figure 2.

**Figure 3:**
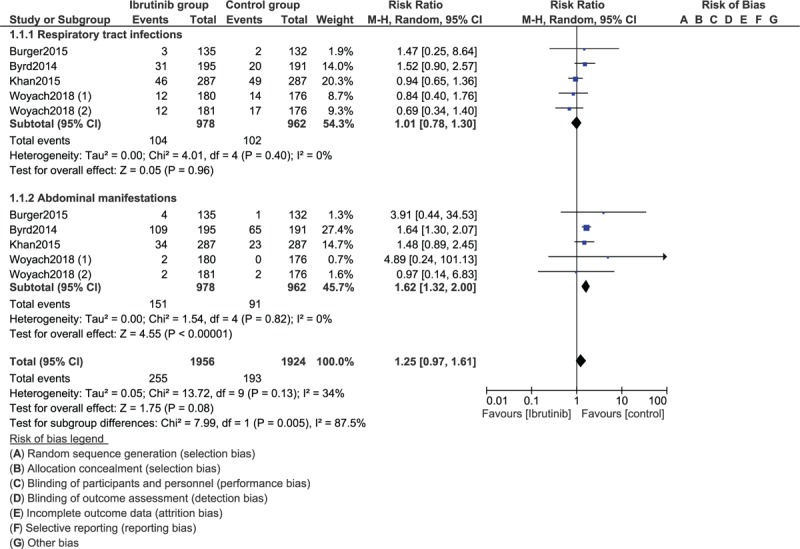
Forest plot demonstrating the adverse drug events observed with ibrutinib versus the control group for the treatment of patients with chronic lymphocytic leukemia (Part II).

The risk for respiratory tract infection was also similarly manifested (RR: 1.01, 95% CI: 0.78–1.30; *P* = .96). However, ibrutinib was associated with a high risk of abdominal manifestations in comparison to the control group (RR: 1.62, 95% CI: 1.32–2.00; *P* = .00001) as shown in Figure 3.

**Figure 4:**
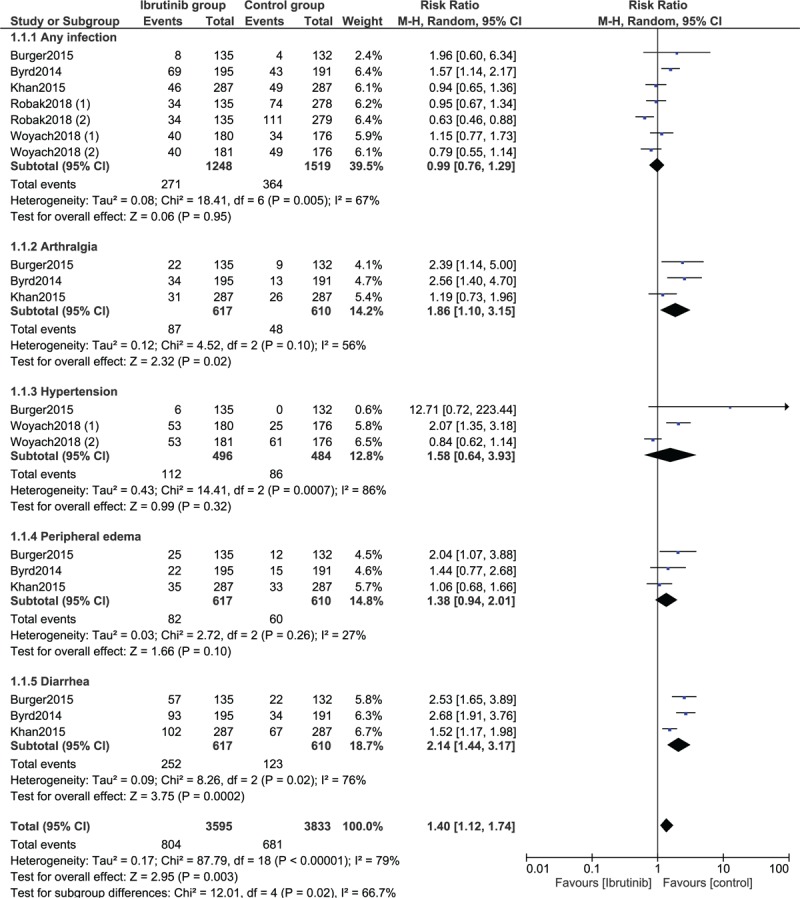
Forest plot demonstrating the adverse drug events observed with ibrutinib versus the control group for the treatment of patients with chronic lymphocytic leukemia (Part III).

The risk for diarrhea was also significantly higher in the ibrutinib group (RR: 2.14, 95% CI: 1.44–3.17; *P* = .0002) as shown in Figure 4. Risks for other adverse drug events were as followed: Any infection (RR: 0.99, 95% CI: 0.76–1.29; *P* = .95), arthralgia (RR: 1.86, 95% CI: 1.10–3.15; *P* = .02), hypertension (RR: 1.58, 95% CI: 0.64–3.93; *P* = .32), and peripheral edema (RR: 1.38, 95% CI: 0.94–2.01; *P* = .10) as shown in Figure 4.

The main results were listed in Table 5.

**Table 5:**
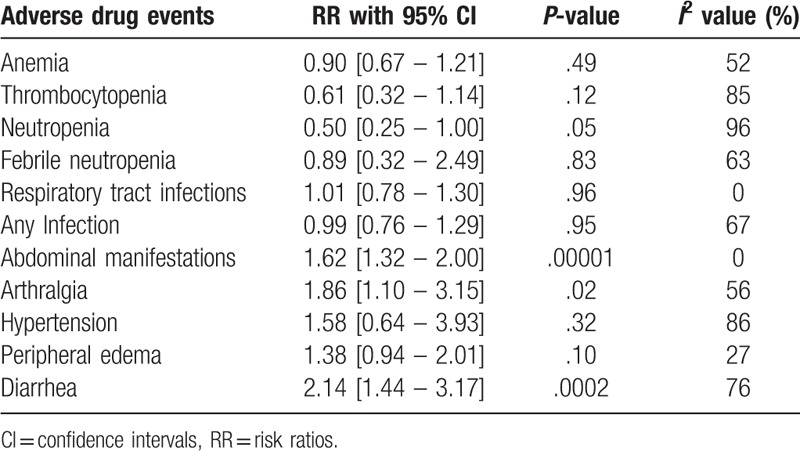
Results analyzing the adverse drug events.

### 3.6 Sensitivity analysis and publication bias

Sensitivity analysis was carried out by a method of exclusion.

When excluding study Byrd2014 (RR: 0.42, 95% CI: 0.19–0.94; *P* = .03) and study Khan 2015 (RR: 0.44, 95% CI: 0.22–0.89; *P* = .02), the risk of neutropenia was significantly lower with ibrutinib. Arthralgia was not significantly different when study Burger 2015 (RR: 1.71, 95% CI: 0.81–3.62; *P* = .16), and study Byrd 2014 (RR: 1.60, 95% CI: 0.81–3.13; *P* = .17) were excluded.

For the remaining outcome analyses, consistent results were obtained throughout.

**Figure 5:**
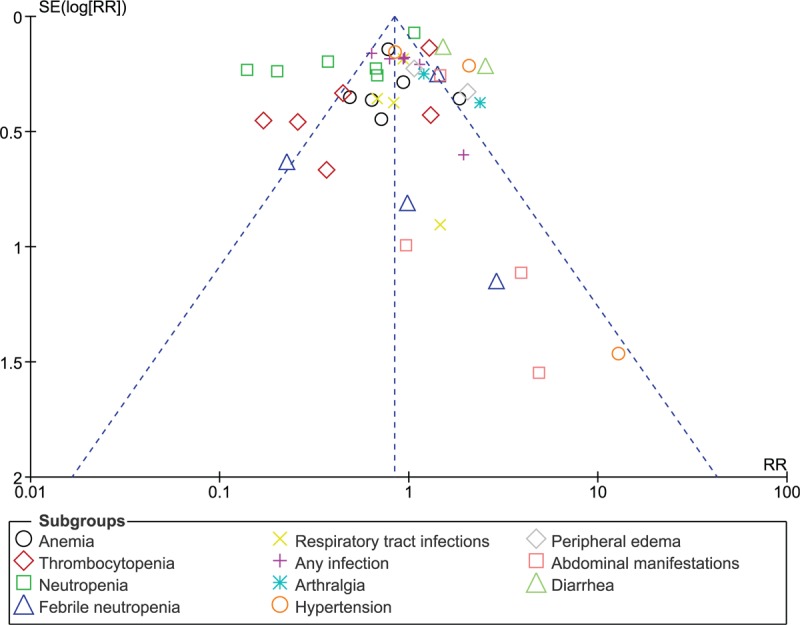
Funnel plot showing publication bias.

Also, minimal publication bias was observed as illustrated in Figure 5.

## 4 Discussion

Ibrutinib was not associated with significantly higher adverse drug events including anemia, neutropenia, and thrombocytopenia compared to its control group for the treatment of patients with CLL. However, this current analysis showed a significantly higher risk of abdominal manifestations with the use of ibrutinib in these patients with CLL.

A recent meta-analysis of randomized controlled trials demonstrating the efficacy and safety of B-cell receptor signaling pathway inhibitors in relapsed/refractory CLL and involving 1866 participants showed these agents to increase the risk of grade 3 and grade 4 adverse events as well as increase the risk of serious adverse events.^[17]^ However, this current result was different in the way that our study consisted of a larger number of participants who were treated with ibrutinib (1113 participants in this current analysis vs 969 participants on ibrutinib for the other meta-analysis). In addition, the effect of idelalisib might have dominated that analysis since 3 out of 5 trials involved the use of idelalisib. Only 2 studies were based on ibrutinib whereas our meta-analysis involved strictly 5 studies with ibrutinib.

Long term (5 years) safety of ibrutinib was recently studied in a population of elderly CLL participants.^[18]^ Even though the adverse drug events manifested more during the first year period of treatment, acceptable tolerability was observed over time. Also, the onset of grade ≥3 cytopenias, including thrombocytopenia and neutropenia were reduced over time. In our current analysis, this decrease in cytopenia was not observed since in this study, we only compared ibrutinib versus a control group and we did not compare baseline versus follow-up outcomes including cytopenia ≥grade 3.

Cases from a first named patient program in Indian CLL participants in a real-world setting also showed ibrutinib to be effective therapeutically.^[19]^ The safety side of this drug was also not questionable. In their research, the authors demonstrated that no grade 3 to 4 or other serious adverse drug events occurred. Dose reduction was also not required, and their study did not report any participant discontinuing treatment due to adverse drug events. This drug might become the physician's drug of choice for the treatment of CLL.^[20]^

A systematic review and network meta-analysis based on the front line treatment of patients with CLL demonstrated positive and superior benefit of ibrutinib (efficacy and safety) including progression-free and overall survival for the treatment of patients with CLL.^[17]^ Another indirect comparison also showed ibrutinib to significantly benefit patients with CLL in comparison to bendamustine.^[21]^

Ibrutinib (28 day cycles of once daily 420 mg) has also been compared with ibrutinib + rituximab (375 mg/m^2^) for the treatment of CLL.^[22]^ Even though remission was faster and significantly lower residual disease levels were achieved with the combination of drugs, a study showed ibrutinib as a single agent to be the actual standard treatment for patients with CLL.

Nevertheless, other recent studies have shown ibrutinib to have a poor response and prognosis in patients with co-morbidities.^[23]^ But, a decreased dosage of the drug might apparently be effective; however, this present study did not focus on the dosage of ibrutinib. In addition, another United Kingdom based study on the cost-effectiveness of ibrutinib showed the latter to be expensive in comparison to other drugs for the treatment of CLL.^[24]^ An adequate discount would be required to achieve cost-effectiveness.^[25]^

A retrospective study of the French innovative leukemia organization also demonstrated the outcomes of CLL patients who, during treatment period, switched from ibrutinib to other alternate kinase inhibitors.^[26]^ The efficacy and toxicity of ibrutinib have further been demonstrated in the Polish Adult Leukemia Group.^[27]^ This drug should further be studied for any serious cardiovascular side effects.^[28,29]^ However, further additional data would be required to better understand the relationship between ibrutinib and cardiovascular effects including atrial fibrillation.^[30]^

## 5 Limitations

The total number of participants were less and this might be a limitation of this analysis. Second, the number of studies was limited during the subgroup assessment of adverse drug events. This might have affected the main analysis. Moreover, the treatment duration was not similar for each study. In addition, different drugs were used in the control group (chlorambucil, ofatumumab, fludarabine, bendamustine, and rituximab) and this might potentially have affected the results of this analysis. In addition, the heterogeneity level was high among several subgroups. A high level of heterogeneity was observed during the assessment for adverse outcomes such as arthralgia, hypertension, thrombocytopenia, neutropenia, any infection, and diarrhea. Several factors including selection bias, language bias, and other confounding factors might have contributed to this increase in heterogeneity. Moreover, the dosage of the drugs was also neglected since dosage was not reported in the original articles and this might as well be considered as a limitation of this study. In addition, it is well known that the use of ibrutinib is often associated with bleeding events and major hemorrhages especially in older patients thus restricting its use in some cases. However, these important adverse events were not assessed in this present study since these outcomes were not reported as adverse drug events in the original papers. Hence, this might also be a major drawback of this analysis and partly limit its clinical relevance.

## 6 Conclusions

During the treatment of CLL, Ibrutinib was not associated with significantly higher risks of anemia, thrombocytopenia or neutropenia compared to the control group. However, abdominal manifestations were significantly higher with ibrutinib. Advanced phase trials should further confirm this hypothesis.

## Author contributions

**Conceptualization:** Yanhua Zhou, Hongtao Lu, Meifeng Yang, Chenhong Xu.

**Data curation:** Yanhua Zhou, Hongtao Lu, Meifeng Yang, Chenhong Xu.

**Formal analysis:** Yanhua Zhou, Hongtao Lu, Meifeng Yang, Chenhong Xu.

**Funding acquisition:** Yanhua Zhou, Hongtao Lu, Meifeng Yang, Chenhong Xu.

**Investigation:** Yanhua Zhou, Hongtao Lu, Meifeng Yang, Chenhong Xu.

**Methodology:** Yanhua Zhou, Hongtao Lu, Meifeng Yang, Chenhong Xu.

**Project administration:** Yanhua Zhou, Hongtao Lu, Meifeng Yang, Chenhong Xu.

**Resources:** Yanhua Zhou, Hongtao Lu, Meifeng Yang, Chenhong Xu.

**Software:** Yanhua Zhou, Hongtao Lu, Meifeng Yang, Chenhong Xu.

**Supervision:** Yanhua Zhou, Hongtao Lu, Meifeng Yang, Chenhong Xu.

**Validation:** Yanhua Zhou, Hongtao Lu, Meifeng Yang, Chenhong Xu.

**Visualization:** Yanhua Zhou, Hongtao Lu, Meifeng Yang, Chenhong Xu.

**Writing – original draft:** Yanhua Zhou, Hongtao Lu.

**Writing – review and editing:** Yanhua Zhou, Hongtao Lu.
